# Psychometric properties of the Autism-Spectrum Quotient in both clinical and non-clinical samples: Chinese version for mainland China

**DOI:** 10.1186/s12888-016-0915-5

**Published:** 2016-07-07

**Authors:** Long Zhang, Yaoting Sun, Fangfang Chen, De Wu, Jiulai Tang, Xiaopeng Han, Jianguo Ye, Kai Wang

**Affiliations:** Department of Neurology, The First Affiliated Hospital of Anhui Medical University, Hefei, China; Collaborative Innovation Center for Neuropsychiatric Disorders and Mental Health, Anhui Medical University, Hefei, China; Department of Children Rehabilitation, The First Affiliated Hospital of Anhui Medical University, Hefei, China; Department of Psychology of Anhui provincial hospital, Hefei, China; Psychological Consultation Center of Anhui Medical University, Hefei, China

**Keywords:** Autism spectrum disorders, Autism-spectrum quotient, Broader autism phenotype, Obsessive-compulsive disorder, Psychometric properties, Schizophrenia

## Abstract

**Background:**

The Autism-Spectrum Quotient (AQ) is widely used to quantify autistic traits, which have been evaluated in the parents of individuals with autism spectrum disorders (ASD) and in the general population. This paper’s objective was to investigate the AQ's psychometric properties of the Chinese version for mainland China and to establish whether the pattern of sex differences in the quantity of autistic traits exists. We also examined the usefulness of the AQ in differentiating between individuals with ASD, schizophrenia (SCH), obsessive-compulsive disorder (OCD) and healthy controls (HC).

**Methods:**

In this study, the psychometric properties of the AQ were assessed in 1037 parents of children with ASD and in 1040 parents of typically developing children (TDC). Additionally, 32 participants with ASD, 37 patients with SCH, 38 OCD patients and 38 healthy controls (matched for age, gender and IQ) were assessed with the AQ.

**Results:**

The internal consistency and test-retest reliability of the AQ and AQ subscales were within an acceptable range. Parents of ASD children scored higher than TDC parents on total AQ and AQ subscales, and TDC parents scored more than parents of ASD children on 2 items of 50. Fathers scored higher than did mothers on total AQ and four subscales, with the sole exception being the subscale attention to detail. The total AQ score of the ASD group was higher than that of the SCH, OCD and HC groups, and the total AQ score of the HC group was significantly lower than that of the SCH and OCD groups, with no differences being observed between the SCH and OCD groups.

**Conclusions:**

The Mandarin AQ demonstrated promising psychometric properties and was a reliable instrument for quantifying autistic traits in both clinical and non-clinical samples in mainland China.

## Background

Autism spectrum disorders (ASD) are a group of disorders characterized by impairments in maintaining reciprocal interaction and communication with others and the presence of narrow interests and stereotyped patterns of behavior and activities [1]. More recently, a quantitative, dimensional reconceptualization of ASD in the general population has been proposed [2]. In other words, the concept of the autistic spectrum, originally conceived as a gradient of severity within the clinical range, has been extended to a continuum of autistic traits in the general population [3–5]. This change suggests that typically developing individuals may display autistic traits that vary in both the degree of severity and number [6]. These traits, known as the Broader Autism Phenotype (BAP), have been examined in the relatives of individuals with ASD and in the general population [7–9]. In a continuum of severity of autistic traits in the general population, the BAP is generally considered to be a subclinical set of characteristics that is milder but qualitatively similar to the diagnosed autism phenotype [10, 11].

One of the most widely used quantitative measures of BAP is the Autism-Spectrum Quotient (AQ). The AQ is a self-report screening instrument for measuring the severity of autistic traits across five subscales (social skills, communication, attention to detail, attention switching and imagination) in both the general population and the autism spectrum community [3]. The 50-item questionnaire has good cross-cultural stability [11–14] and demonstrates consistent results across different age groups [15–17]. The AQ exhibits its own advantages compared with other self-administered measures of BAP; for example, the AQ has been shown to distinguish between individuals with high-functioning ASD and individuals with other psychiatric disorders [18]. These characteristics have demonstrated that the AQ is a reliable instrument for quantifying the BAP, has been widely used with relatives of individuals with ASD, and has the ability of screening for autistic traits in the general population [5, 7, 11, 19]. The AQ score is related to individuals’ performance on gaze-oriented attention to happy faces [20] and on the global integration of closed contours [21], has been found to be linked with white matter fiber tract [22] and has been associated with white matter volume in the posterior superior temporal sulcus [23].

It is particularly worth noting that Baron-Cohen et al. have found sex differences in the general population. The mean total AQ score was higher in males than in females [3]. Similar sex differences in the AQ were found in studies conducted in the Netherlands [8], Scotland [9], Italy [5] and Poland [13]. The incidence of autism was much higher in males than in females [24]. The exploration of sex differences in the severity of autistic traits in different groups of people and cultural contexts may contribute to a deeper understanding of the association between autism and gender [13].

Existing evidence suggests that ASD and psychosis spectrum disorders share the clinical symptoms and manifestations [25]. Longitudinal studies have found that children having a greater severity of early autistic traits are more likely to have psychotic experiences in early adolescence [25, 26]. In addition, individuals with ASD were more likely to report schizophrenic symptoms, and schizophrenia (SCH) patients were more likely to report autistic symptoms [27–30]. Similarly, both ASD and obsessive-compulsive disorder (OCD) have common features, such as obsessional interests and repetitive or stereotypic behaviors. Furthermore, certain vulnerability genes may prove to be generalist genes, influencing the phenotypic expression of both ASD and OCD [31]. Individuals with ASD were more likely to show obsessive-compulsive traits, and patients with OCD were more likely to show autistic symptoms [32, 33].

Regarding the studies noted above, determining whether the symptoms of individuals with ASD resemble those with SCH or OCD is necessary, and identifying the similarities and differences between ASD and the two other disorders is vitally important [34]. Therefore, there is a growing demand for research on investigating how ASD can be effectively and sufficiently differentiated from other psychiatric disorders. The AQ is a widely used screening instrument that can be used to distinguish validly between high functioning adults with autism and individuals with other psychiatric disorders [18]. Previous research has found that, in the autistic traits continuum, subjects with autism lie on one end and neurotypical subjects lie on the other end; the individuals with SCH or OCD can be placed approximately in the middle of this continuum [28, 35].

To date, no standardized and brief measures that could be practical in ASD screening purposes have been made available in mainland China. The adaptation and validation of the AQ for the Mandarin Chinese-speaking population is important and necessary for both research and ASD screening purposes [13]. Prior studies from Western cultures have shown that the parents of ASD children score significantly higher than do the parents of TDC children on the AQ, and men tend to have significantly higher scores than women on the AQ. These general patterns were replicated in Japan, India and Malaysia [12, 36], which suggests that these patterns are stable and are independent of cultural influences, thus providing strong support for the validity of the AQ as an instrument for use in Eastern cultures. However, there are cultural nuances for certain autism-related behaviors, as described by the AQ. For instance, certain behaviors that are considered to be related to attention in Western cultures may actually have a social significance in Eastern cultures. In this study, we wanted to investigate the psychometric properties of the AQ and to examine whether the patterns noted above could be replicated in mainland Chinese populations.

In addition to a group with parents of ASD and a group with parents of typically developing children (TDC), this study included a sample of three different patient groups (a group with ASD, a group of patients with SCH and a group with OCD) and a healthy control group. In accordance with prior researches, we predicted that the AQ scores in these patient groups will be higher than the general population mean. Extremely high AQ scores were expected to be specific to individuals with ASD.

Primarily, the characteristics of the Mandarin Chinese AQ, including the internal consistency, the test-retest reliability and the discriminating power of items, were examined in a large sample of parents of ASD children and TDC parents. In accordance with the findings from previous studies, we hypothesized: 1) AQ scores to be continuously distributed in these two groups of parents; 2) higher AQ scores in parents of ASD children than in TDC parents; and 3) significantly higher mean AQ scores in males compared with females.

## Methods

### Participants and procedure

The parents groups enrolled a group with parents of autism spectrum disorders (ASD) and a group with parents of typically developing children (TDC), including 1037 parents of ASD children (515 fathers and 522 mothers) and 1040 parents of TDC (525 fathers and 515 mothers). This study included 467 couples with ASD children and 489 couples with TDC. The remainder of fathers and mothers belonged to different families (parents of ASD children: 48 fathers and 55 mothers; TDC parents: 36 fathers and 26 mothers). There was no significant difference between the mean age of the parents of the ASD group (35.51 ± 4.56 years) and those of the control TDC (35.86 ± 4.48 years; *t* = − 1.78, *P* = 0.075). The mean age of the fathers of the ASD probands was 35.53 ± 4.51 years, and that of the mothers was 35.48 ± 4.62 years. The mean age of the fathers of the TDC was 35.82 ± 4.52 years, and that of the mothers was 35.90 ± 4.44 years. There was no significant difference between the mean ages of fathers (*t* = − 1.05, *P* = 0.296) and mothers (*t* = − 1.47, *P* = 0.141). There was no significant difference between the educational level of the parents of ASD children (12.51 ± 2.69 years) and those of TDC (12.62 ± 2.68 years; *t* = − 0.93, *P* = 0.354).

Parents with ASD children were contacted through institutions and organizations that provide assistance to ASD children in mainland China, as well as medical institutions that provide diagnoses of ASD. ASD families included parents of children diagnosed as having Autism Disorder (AD), Asperger Syndrome (AS) and Pervasive Developmental Disorder Not Otherwise Specified (PDD-NOS) based on the DSM-IV-TR diagnostic criteria for AD, AS and PDD-NOS [1]. Chinese adaptations of such tools as the Autism Diagnostic Interview-Revised [37] and the Autism Diagnostic Observation Schedule [38] are not available to date in mainland China. To increase the accuracy of the subjects’ qualification to the ASD group, all of the participants were diagnosed for the presence of ASD according to the DSM-IV-TR by two independent child psychiatrists with experience in the assessment and treatment of ASD. Parents were included in the TDC parents group in terms of two criteria: they did not have a child with a relevant diagnosis, including ASD, intellectual disability or any significant behavioral problems, and they had no any psychiatric disorders or serious medical illness.

In addition, the present study included three different patient groups and a healthy controls (HC) group. The ASD group was made up of 32 individuals (26 males and 6 females; mean age: *M* = 19.41, *SD* = 3.88) with Asperger syndrome or high-functioning autism. The schizophrenia (SCH) group was comprised of 37 individuals (30 males and 7 females; mean age: *M* = 20.95, *SD* = 3.67). The obsessive-compulsive disorder (OCD) group totaled 38 individuals (31 males and 7 females; mean age: *M* = 21.29, *SD* = 3.15). The SCH patients and OCD patients were recruited from the Mental Health Center of Anhui Province. Patients were included if they 1) met the DSM-IV-TR diagnostic criteria for SCH or OCD, 2) did not meet any other DSM-IV-TR axis I diagnosis, and 3) had never been treated with any psychiatric medication. The HC group was comprised of 38 individuals (30 males and 8 females; mean age: *M* = 21.32, *SD* = 3.32) from college students and the local community who had not been diagnosed with any psychiatric disorders or serious medical illness. They were recruited as HC by advertisements and leaflets or by word of mouth. IQ was tested with the standardized Raven test [39] for the four groups (mean IQ: ASD, *M* = 102.3, *SD* = 14.4; SCH, *M* = 106.6, *SD* = 16.8; OCD, *M* = 103.2, *SD* = 11.1; HC, *M* = 108.9, *SD* = 12.9). Among the four groups, there were no significant differences in age (*F* = 2.24, *P* = 0.087), sex ratio (*χ*^2^ = 0.10, *P* = 0.991) or IQ (*F* = 1.71, *P* = 0.168).

### The Mandarin Chinese Autism-Spectrum Quotient

The Autism-Spectrum Quotient (AQ) was translated from English into Mandarin Chinese upon obtaining the consent of Prof. Simon Baron-Cohen. To maintain the meaning of words and sentences between English and Mandarin Chinese, a back-translation was conducted as follows: first, the AQ was translated into Mandarin Chinese by a native Chinese speaker. Next, a native English speaker who was not familiar with the AQ translated it back into English. The original English and back-translated versions were compared by a native English speaker. Discrepancies were revised to more accurately express the intent of the wording in the original version. Therefore, the Mandarin Chinese AQ could be considered linguistically equivalent to the original English version (the Mandarin Chinese version of the questionnaire is obtainable from the first author upon request).

This study used the original AQ [3], a self-report questionnaire that consists of 50 descriptive statements that assess personal behaviors, habits and preferences pertinent to the clinical manifestation of ASD. All items are equally divided into five subscales, each with 10 items. Every response that is characteristic for autism is scored “1” if “definitely agree” or “slightly agree”, and otherwise “0” if “definitely disagree” or “slightly disagree”. However, in accordance with the precedent of previous studies [8, 9, 19, 40], our study employed a continuous (4-point Likert) scale (ranging from 1 to 4 for items portraying autistic feature: “definitely agree” scored 4 points, “slightly agree” scored 3 points, “slightly disagree” scored 2 points, and “definitely disagree” scored 1 points, and the scale was inverted for the opposite items). The use of all of the response option choice information reserves more information regarding the subjects’ responses than the 0/1 scoring. This use also maximizes scale reliability and validity coefficients and increases the likelihood of obtaining a better approximation of continuous distribution. All item scores are summed, and higher scores depict closer proximity to prototypical ASD.

### Ethics statement

Informed consents were obtained from all the participants. For subjects under 18 years of age, their parents also signed the informed consent form. The study was executed in agreement with the Declaration of Helsinki and was approved by the Ethics Committee at Anhui Medical University.

## Results

### Distributions of the AQ scores

**Fig. 1:**
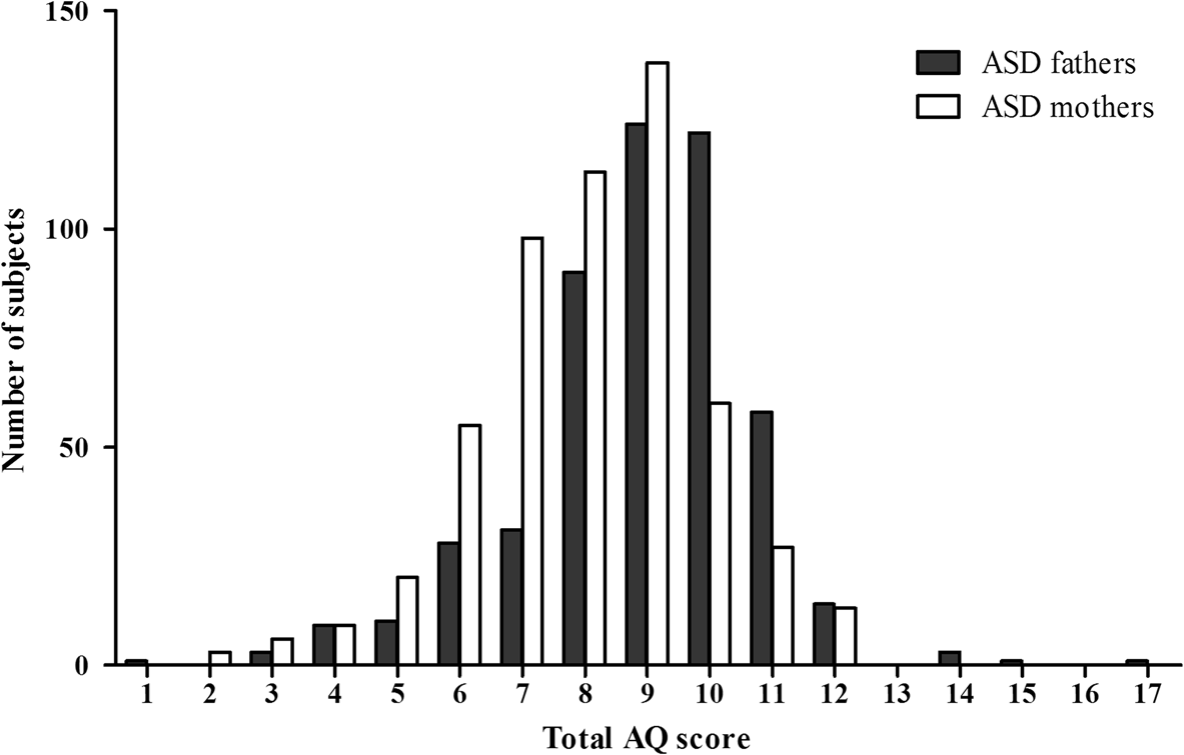
Distributions of total AQ scores in parents of ASD children. Each number corresponds to a total AQ scores range: 1, 71–75; 2, 76–80; 3, 81–85; 4, 86–90; 5, 91–95; 6, 96–100; 7, 101–105; 8, 106–110; 9, 111–115; 10, 116–120; 11, 121–125; 12, 126–130; 13, 131–135; 14, 136–140; 15, 141–145; 16, 146–150; 17, 151–155

**Fig. 2:**
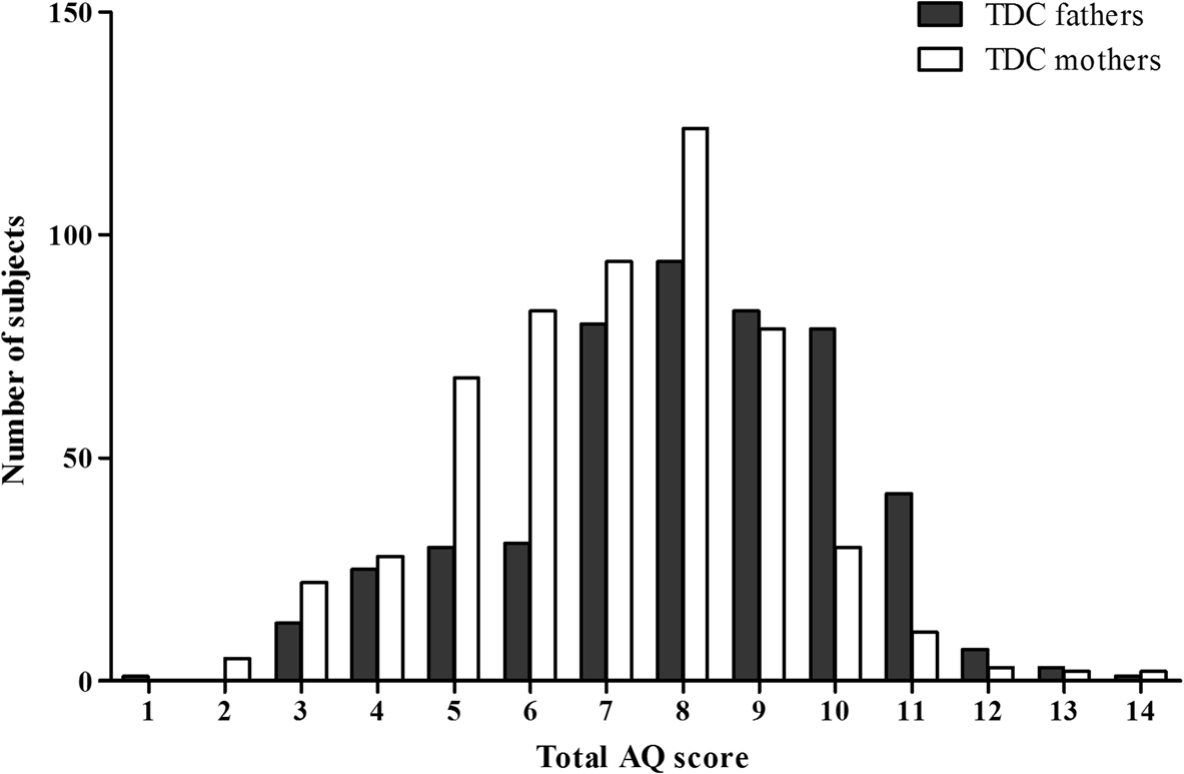
Distributions of total AQ scores in TDC parents. Each number corresponds to a total AQ scores range: 1, 71–75; 2, 76–80; 3, 81–85; 4, 86–90; 5, 91–95; 6, 96–100; 7, 101–105; 8, 106–110; 9, 111–115; 10, 116–120; 11, 121–125; 12, 126–130; 13, 131–135; 14, 136–140

Table 1 shows the mean (and SD) scores on total AQ and AQ subscales for parents of ASD children and TDC parents. Higher scores indicate more autistic traits. TDC parents had a lower Total AQ score compared to parents of ASD children; this difference was significant, *t* = 10.89, *P* < 0.001. The distributions of total AQ scores in ASD fathers versus ASD mothers, and TDC fathers versus TDC mothers, are presented in Fig. 1 and Fig. 2, respectively. It’s worth mentioning that intraclass correlation for total AQ scores between fathers and mothers was statistically significant in ASD family (*r* **=** 0.14, *p* **=** 0.002), suggestive of the presence of assortative mating. There was no significant correlation for total AQ scores between fathers and mothers in TDC family (*r* **=** 0.04, *p* **=** 0.354).

**Table 1:** Mean (and SD) scores on the total AQ and AQ subscales for parents of ASD children and TDC parents

	Total AQ	Social skills	Attention switching	Attention to detail	Communication	Imagination
ASD patients						
Total	110.4 (9.22)	22.3 (4.07)	23.1 (3.04)	24.3 (4.09)	20.9 (3.71)	19.8 (3.70)
Fathers	112.7 (9.04)	22.6 (4.21)	23.8 (3.01)	24.4 (4.08)	21.4 (3.81)	20.5 (3.71)
Mothers	108.3 (8.88)	22.0 (3.93)	22.5 (2.91)	24.2 (4.11)	20.4 (3.57)	19.3 (3.60)
TDC patients						
Total	105.6 (10.54)	21.0 (4.08)	22.4 (3.46)	23.1 (3.89)	19.9 (3.76)	19.2 (3.59)
Fathers	108.1 (10.68)	21.7 (4.16)	23.1 (3.51)	23.1 (4.09)	20.6 (3.62)	19.7 (3.78)
Mothers	103.4 (9.91)	20.4 (3.91)	21.8 (3.30)	23.1 (3.70)	19.4 (3.80)	18.7 (3.34)

*AQ* autism-spectrum quotient; *ASD* autism spectrum disorder; *TDC* typically developing children

### Group and sex differences on the AQ

Table 2 shows the AQ and the five subscales scores in the parents groups. The *F* values and partial η^2^ values generated by separate ANOVAs, with between-subject factor of group and within-subject factor of sex are also shown. A main effect for group was found on total AQ and five subscales, where the parents of ASD children scored higher than the TDC parents (all *p*s < 0.001). A main effect for sex was found in total AQ and four subscales, where fathers scored higher than mothers (all *p*s < 0.001). The only exception was attention to detail, where the effect of sex was not significant. Group by sex interactions did not approach significance (all *p*s > 0.05).

**Table 2:** Two-way ANOVA group × sex over the total AQ and AQ subscales in the parents groups

	Group main effect	Group effect size	Sex main effect	Sex effect size	Group x sex interaction	Group x sex effect size
	F (2073)	Partial η^2^	F (2073)	Partial η^2^	F (2073)	Partial η^2^
Total	122.64*	0.056	115.67*	0.053	0.18	<0.001
Social skills	48.27*	0.023	24.91*	0.012	3.29	0.002
Attention switching	25.37*	0.012	92.37*	0.043	0.05	<0.001
Attention to detail	46.87*	0.022	0.26	<0.001	0.37	<0.001
Communication	31.30*	0.015	47.19*	0.022	0.38	<0.001
Imagination	16.32*	0.008	49.72*	0.023	0.44	<0.001

*AQ* autism-spectrum quotient; η^2^, eta squared

*p < 0.001

### Internal consistency and test-retest reliability

The internal consistency of the AQ was assessed using Cronbach’s *α* coefficient in the parents groups. As shown in Table 3, the internal consistency of the total AQ and AQ subscales were satisfactory, and the Cronbach’s *α* coefficients were moderate to high in both samples. To measure the test-retest reliability a second AQ measurement was conducted on a group of 176 parents of ASD children and a group of 182 TDC parents after one month. Table 3 shows that scores from the first and second AQ measurements did not differ statistically (all *p*s ≥ 0.25 for parents of ASD children and all *p*s ≥ 0.174 for TDC parents). Additionally, the first AQ scores were strongly correlated with the second AQ scores, and the Pearson *r* coefficients were as follows (parents of ASD children/TDC parents): *r* = 0.79/0.89 for total AQ, *r* = 0.75/0.85 for social skills, *r* = 0.44/0.62 for attention switching, *r* = 0.68/0.81 for attention to detail, *r* = 0.67/0.70 for communication and *r* = 0.42/0.70 for imagination.

**Table 3:** Test–retest reliability and internal consistency of the AQ

	Test–retest reliability				Internal consistency
	1st [M (SD)]^a^	2nd [M (SD)]^a^	*t* ^a^	*p* ^a^	Cronbach *α* ^a^
Total	109.4 (8.75)/105.4 (9.55)	109.6 (8.12)/106.2 (8.54)	−0.20/-0.79	0.845/0.432	0.817/0.806
Social skills	23.3 (4.48)/21.7 (4.16)	23.4 (4.06)/21.7 (3.63)	−0.27/-0.15	0.784/0.883	0.743/0.760
Attention switching	24.1 (2.93)/23.4 (2.98)	23.7 (2.51)/23.2 (2.56)	1.15/0.70	0.250/0.485	0.640/0.657
Attention to detail	22.5 (4.00)/21.9 (4.19)	22.6 (3.71)/22.0 (3.75)	−0.18/-0.26	0.857/0.792	0.673/0.694
Communication	20.1 (3.51)/19.4 (3.47)	20.4 (3.30)/19.8 (3.05)	−0.39/-1.36	0.696/0.174	0.765/0.756
Imagination	19.3 (3.01)/19.1 (3.38)	19.5 (2.87)/19.4 (3.22)	−0.54/-0.91	0.588/0.366	0.622/0.619

*AQ* autism-spectrum quotient

^a^The former is the result from ASD parents (*n* = 176). The latter is the result from TDC parents (*n* = 182)

### Discriminating power of items

To estimate the discriminating power of items in the AQ, the percentages of parents with ASD children and parents with TDC scoring 3 or 4 points (indicating more autistic features) in each AQ item were calculated. As shown in Table 4, TDC parents scored higher than parents of ASD children on only 2 items out of 50 (item 18 and item 30). As for all the other items, the percentages of parents of ASD children were higher than TDC parents. This finding indicated that the discriminating power of the majority of items was acceptable and that the AQ was an useful questionnaire in distinguishing parents of ASD children from TDC parents in this study.

**Table 4:** Percentages of parents of ASD children and TDC parents who scored 3 or 4 in individual items of the AQ

AQ Item	ASD (*n* = 32)	TDC (*n* = 38)	AQ Item	ASD (*n* = 32)	TDC (*n* = 38)
1	50.00	5.26	26	78.13	42.11
2	71.88	44.74	27	50.00	21.05
3	21.88	0.00	28	56.25	31.58
4	68.75	68.42	29	59.38	42.11
5	65.63	44.74	30	56.25	68.42
6	43.75	39.47	31	43.75	5.26
7	43.75	7.89	32	65.63	60.53
8	50.00	21.05	33	56.25	23.68
9	31.25	13.16	34	34.38	15.79
10	53.13	15.79	35	31.25	18.42
11	81.25	36.84	36	53.13	26.32
12	68.75	68.42	37	46.88	39.47
13	78.13	28.95	38	90.63	21.05
14	71.88	52.63	39	59.38	23.68
15	37.50	36.84	40	53.13	18.42
16	59.38	47.37	41	53.13	13.16
17	71.88	18.42	42	50.00	26.32
18	15.63	15.79	43	73.68	65.63
19	53.13	26.32	44	75.00	26.32
20	34.38	13.16	45	50.00	31.58
21	56.25	38.64	46	87.50	39.47
22	62.50	13.16	47	50.00	5.26
23	53.13	50.00	48	78.13	31.58
24	75.00	47.37	49	56.25	31.58
25	71.88	50.00	50	53.13	13.16

*AQ* autism-spectrum quotient; *ASD* autism spectrum disorder; *TDC* typically developing children

### Differences in the total AQ and AQ subscales between the three patient groups and the HC group

**Fig. 3:**
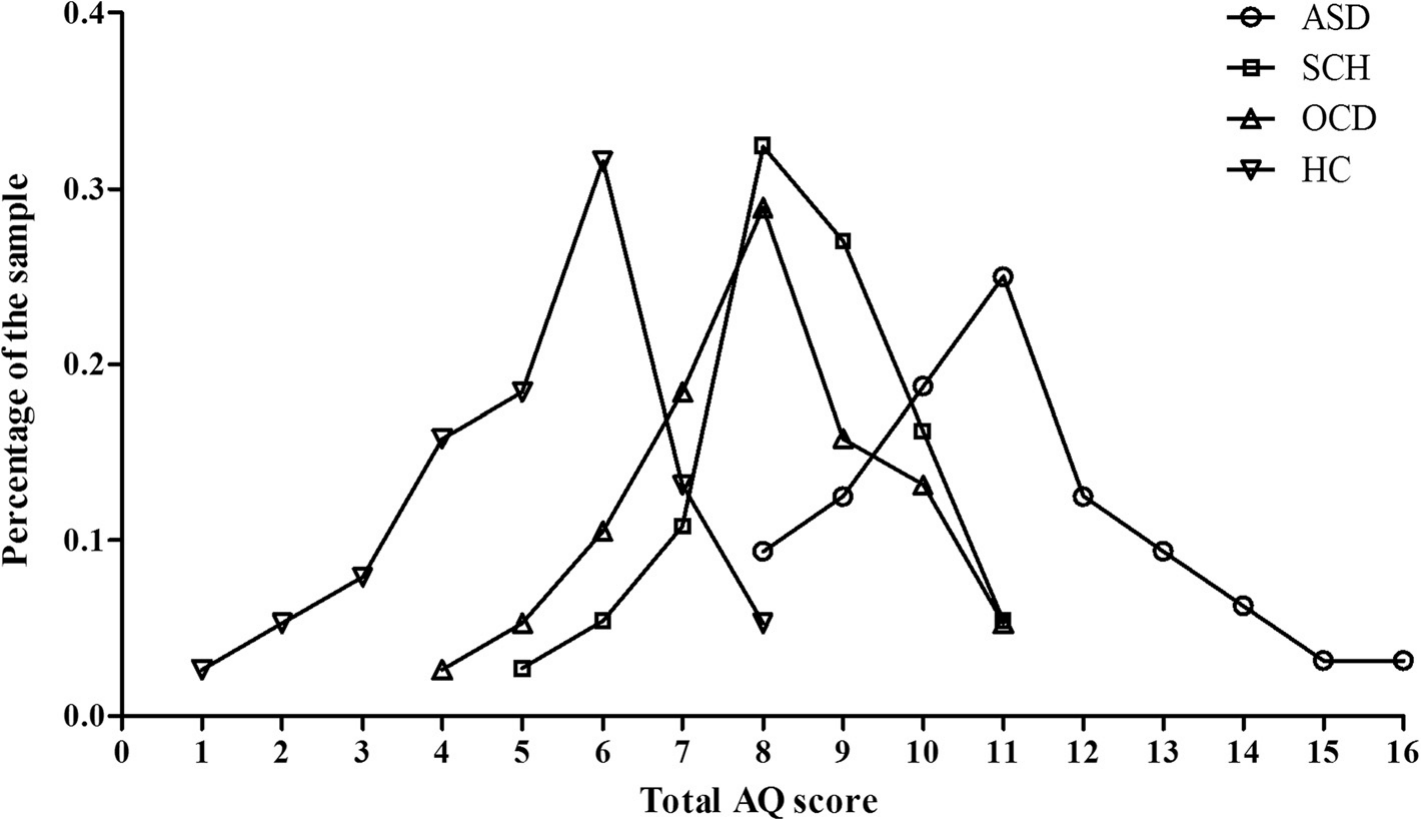
Distributions of total AQ scores in the ASD, SCH, OCD and HC groups. Each number corresponds to a total AQ scores range: 1, 81–85; 2, 86–90; 3, 91–95; 4, 96–100; 5, 101–105; 6, 106–110; 7, 111–115; 8, 116–120; 9, 121–125; 10, 126–130; 11, 131–135; 12, 136–140; 13, 141–145; 14, 146–150; 15, 151–155; 16, 156–160

The total AQ scores and AQ subscales scores for the three patients groups and HC group are presented in Table 5. The distribution of total AQ scores in the four groups are presented in Fig. 3. A one-way analysis of variance with group as the between-subject factor was conducted and showed a statistically significant difference in the total AQ score between the four groups (*F*(3, 141) = 74.21, *p* < 0.001). According to the post-hoc Bonferroni-corrected comparisons, the mean total AQ score of the ASD group was significantly higher than that of the SCH, OCD and HC groups (all *p*s < 0.001). Furthermore, the mean total AQ score of the HC group was significantly lower than that of the SCH and OCD groups (all *p*s < 0.001). However, there was no significant difference in the total AQ score between the SCH and OCD group (*p* = 1.000).

**Table 5:** Mean (and SD) scores on the total AQ and AQ subscales for ASD, SCH, OCD and HC groups

	Total AQ	Social skills	Attention switching	Attention to detail	Communication	Imagination
ASD	133.4 (10.01)	28.3 (4.74)	26.5 (4.24)	26.7 (4.50)	27.6 (4.31)	24.4 (2.92)
SCH	120.5 (6.80)	26.2 (3.11)	22.9 (3.69)	23.3 (4.18)	25.4 (4.36)	22.7 (3.11)
OCD	118.3 (8.32)	23.7 (3.45)	24.9 (2.54)	26.5 (5.05)	21.3 (3.58)	21.8 (3.13)
HC	103.5 (8.54)	20.6 (4.11)	22.5 (3.06)	21.9 (3.58)	18.3 (3.60)	20.2 (2.86)
*F* (3,141)	74.21	25.96	10.29	10.88	38.30	11.68
*p*-Value	<0.001	<0.001	<0.001	<0.001	<0.001	<0.001

*AQ* autism-spectrum quotient; *ASD* autism spectrum disorder; *SCH* schizophrenia; *OCD* obsessive-compulsive disorder

All of the AQ subscales score between the four groups significantly differed (all *p*s < 0.001). According to the post-hoc Bonferroni-corrected comparisons, the ASD group scored significantly higher compared to the SCH group on the subscales of attention switching (*p* < 0.001) and attention to detail (*p* = 0.010). In comparison with the OCD group, the ASD group scored significantly higher on the subscales of social skills, communication and imagination (all *p*s < 0.01). In comparison with the HC group, the ASD group scored significantly higher on all five subscales (all *p*s < 0.001).

In comparison with the HC group, the SCH group scored significantly higher on the subscales of social skills, communication and imagination (all *p*s ≤ 0.01). Compared with the HC group, the OCD group scored significantly higher on the subscales of social skills, attention switching, attention to detail and communication (all *p*s ≤ 0.05). Finally, the SCH group reported significantly more autistic traits than the OCD group on the subscales of social skills (*p* = 0.045) and communication (*p* < 0.001), whereas the OCD group showed significantly more autistic traits than the SCH group on the subscales of attention switching and attention to detail (all *p*s < 0.05).

## Discussion

First, this study set out to determine the basic psychometric properties of the Mandarin Chinese version of the AQ. Next, we studied the distribution of AQ scores in the parents of ASD children and TDC parents and performed an analysis on sex differences in the AQ. Furthermore, this study examined the usefulness of the AQ in differentiating between individuals with ASD, SCH and OCD.

To obtain a better understanding of the differences in AQ scores within the general population, the distribution of total AQ scores in the parents of ASD children and the TDC parents are graphed (refer to Fig. 1 and Fig. 2, respectively). These two figures illustrated that AQ scores followed a continuous distribution in the parents groups. As found in previous studies [3, 6, 8], AQ scores in the ASD and TDC parents groups had an approximately normal distribution. This study showed that symptom features of the autistic profile in parents of ASD children were not obviously different from that in TDC parents. These findings suggest that the Mandarin Chinese AQ model is fitting for a continuum of autistic expression in agreement with the perspective that autistic traits are part of a broader phenotype, on which features lie along a continuum [6]. Therefore, the quantitative, dimensional reconceptualization of ASD is reflected not only in the general population but also in parents of individuals with ASD.

The analysis of group differences showed that the parents of ASD children scored significantly higher than the TDC parents in the total AQ scores and the subscales. These results are in accordance with most of the previous related studies [5, 11] and extend the earlier finding by Bishop et al. [41], who found that AQ scores differentiate the parents of children with ASD from healthy control parents on two subscales (communication and social skills) in a small sample. To the best of our knowledge, there is one sole study that did not find any significant difference between the parents of children with ASD and control parents [42]. However, this study included 25 parents with autistic children and 25 control parents. Our study provided more valuable data regarding the discriminant power of the AQ obtained from a comparison of the parents of ASD children with the controls matched for sex, age and educational level.

Although previous studies found sex differences in healthy adults [3, 8, 43], it is crucial to determine whether the sex effects play a part in the expression of the BAP phenotype in parents. In our study, the analysis of sex differences revealed that, in the total AQ as well as in four of its subdomains (social skills, communication, attention switching and imagination), fathers scored higher than mothers. The exception to this was the subdomain of attention to detail; in this, there was no sex difference. Similar differences have previously been reported in samples of parents [10] and samples of students [13, 44]. Dawson et al. using the Broader Phenotype Autism Symptom Scale, which is another quantitative assessment of autism symptom-related traits, also found the sex differences [7].

With regard to internal reliability, the findings of our study are similar to those obtained by other researchers in other cultures [13, 18, 45]. Internal consistency was highest for the total AQ, social skills and communication, whereas other subscales demonstrated moderate Cronbach’s α coefficients. It is also worth noting that the lowest Cronbach’s α was found for imagination. These results were in accordance with previous studies in other language samples [5, 12, 13, 18, 43], regardless of the version of the instrument. In at least four existing factor analytic studies, the subscale imagination was never established as a separate subscale [8, 19, 45, 46]. Obviously, the items that develop the subscale imagination load on different subscales of the AQ. Therefore, it is possible that the lowest Cronbach’s α for imagination could be due to the questionable validity of this particular subscale [28].

The results of the item analysis indicated that the discriminating power of most of the items was acceptable. On 2 items of 50 (items 18 and 30), the group of TDC parents scored higher than the group of parents of ASD children, which strongly confirms the value of these items for discriminating between parents of ASD children and TDC parents. However, it should be stressed that, as in this study, the British and French studies [3, 14] have also found that healthy controls scored higher than ASD participants on item 30. This finding may show that item 30 should be improved or revised.

In this study, participants in SCH and OCD groups scored significantly higher on the total AQ than healthy controls but lower than participants with ASD. It appears that the total AQ score follows a continuous distribution in the healthy controls; participants with ASD fall in the upper end of this distribution [28], and SCH and OCD patients obtain scores between the healthy controls’ mean and scores typical for participants with ASD. These results demonstrate that the AQ appears useful for distinguishing the SCH and OCD groups from participants with ASD and healthy controls.

More significantly, there were disorder-specific manifestations on the AQ between the SCH and OCD groups. The results showed that the SCH group reported more problems in social skills and communication compared to the individuals with OCD, as was found in the ASD group compared to the OCD group. This finding means that there is potential symptoms overlap between the ASD and SCH groups, and the symptoms here mainly refer to impairments in social interaction. These findings were in accordance with previous studies that found a high degree of similarity in the social function deficits experienced by individuals with ASD and SCH [47, 48]. Furthermore, our findings revealed that the OCD group reported more problems in attention switching and attention to detail compared to the individuals with SCH, as was found in the ASD group compared to the SCH group. These results imply that ASD and OCD may share attention problems, as was found in another study that suggested attention problems may reflect both symptoms overlap and a common etiological factor underlying ASD and OCD [49].

Several shortcomings of this study should be noted. First, we did not assess the predictive validity of the AQ. Second, certain clinical features such as the severity of symptoms and the symptom dimensions reported by the patients were not addressed in this study. The presence of such clinical features could influence the AQ score as reported by the patients. Ideally, the validity of the AQ should also have been studied by comparing the AQ score with another self-administered questionnaire designed to measure autistic traits. However, there are no other self-reported questionnaires in mainland China; therefore, we could not administer more questionnaires to the participants.

## Conclusions

The results of this current study suggest that the Chinese version of the AQ has good psychometric properties and is a reliable and valid questionnaire to quantify autistic traits in the mainland Chinese population. These results also confirm that the Mandarin Chinese AQ measures BAP in an independent, culturally different population. Future studies are warranted to further determine the optimal cut-off score and to relate AQ scores to neurological or genetic differences.

## Abbreviations

AD, autism disorder; AQ, Autism-Spectrum Quotient; AS, Asperger syndrome; ASD, autism spectrum disorders; BAP, Broader Autism Phenotype; HC, healthy controls; OCD, obsessive-compulsive disorder; PDD-NOS, pervasive developmental disorder not otherwise specified; SCH, schizophrenia; TDC, typically developing children
